# Exploring Polymer-Based Additive Manufacturing for Cost-Effective Stamping Devices: A Feasibility Study with Finite Element Analysis

**DOI:** 10.3390/polym16131894

**Published:** 2024-07-02

**Authors:** Cristian Giolu, Cristina Pupăză, Cătălin Gheorghe Amza

**Affiliations:** Faculty of Industrial Engineering and Robotics, National University of Science and Technology Politehnica Bucharest, 060042 Bucharest, Romania; cristian.giolu@gmail.com (C.G.); cristina.pupaza@upb.ro (C.P.)

**Keywords:** MEX, additive manufacturing, stamping die, bending, deep drawing, sheet metal

## Abstract

This research investigates the feasibility of manufacturing stamping devices using Material Extrusion (MEX) Additive Manufacturing (AM) technology, traditionally fabricated from metal, to reduce production costs and time. This study examines polymer-based devices subjected to Finite Element Analysis (FEA) to evaluate their performance in stamping metal sheets of varying thicknesses. The findings reveal that ABS polymer devices, while demonstrating potential, operate near the material’s limit under compression forces, particularly for sheet thicknesses up to 1 mm. Specifically, differences of 0.7 mm were observed at the connection radii of 0.25 mm sheets and 1.4 mm for 0.5 mm sheets, with angular deviations of 1.5 degrees for 0.25 mm sheets and 4 degrees for 0.5 mm sheets. Additionally, devices made of Nylon were deemed suitable for reduced-thickness sheets (0.25 mm), performing better than those made of ABS. These results suggest that while ABS devices exhibit significant deviations (up to 45 degrees for 1 mm sheets), the method shows promise for small batch production and prototyping. Further optimisation through material enhancements and mechanical improvements is recommended to minimise deformations and enhance precision.

## 1. Introduction

The deep drawing process, a widely used manufacturing technique in industries ranging from the automotive to consumer goods, involves the transformation of flat sheet metal into three-dimensional shapes through the application of mechanical force [1]. Key factors influencing the success of deep drawing include the geometry of the parts involved, material properties such as hardness and surface finish, lubrication, blank holder force, and process parameters such as the draw ratio and strain distribution [2].

Manufacturing deep drawing dies via conventional methods entails significant costs, primarily attributed to materials, labour, and machining processes. The largest portion of the cost is often associated with the materials required for constructing the die, which typically consists of high-strength tool steels or other specialised materials capable of withstanding the high pressures and repetitive stresses involved in the deep drawing process [3,4]. Additionally, skilled labour is necessary for the design, machining, and assembly of the die components, contributing to the overall manufacturing cost [5].

The economies of scale associated with mass production allow the upfront costs of die manufacturing to be spread across a large number of parts, resulting in a lower per-unit cost [3,4,5,6]. This cost efficiency is particularly advantageous for industries that require large quantities of identical or similar parts, such as automotive or appliance manufacturing [4].

Furthermore, the lifespan of a deep drawing die is a critical factor in assessing its cost-effectiveness. With proper maintenance and periodic refurbishment, deep drawing dies can last for a significant number of production cycles, ranging from tens of thousands to hundreds of thousands of parts [2], depending on factors such as material selection, die design, and operating conditions.

Conventionally, the manufacturing of stamping devices is performed using metal, and through a multitude of possible operations, processed materials, and variations in the thickness of the blank, it has led to the development of a wide range of specialised alloys to keep up with mechanical demands [6]. So, choosing the right material becomes a significant aspect, especially in the current industry context, where the cost of production tends to be minimised [7].

However, while deep drawing dies offer significant cost advantages for high-volume production scenarios, their efficiency diminishes for low-batch or bespoke production runs [3,4,5]. In such cases, the upfront costs associated with die manufacturing may outweigh the benefits of mass production economics. The high initial investment in die design and fabrication becomes less financially viable when spread across a limited number of parts, resulting in a higher per-unit cost for each component produced. Furthermore, the extended lifespan of deep drawing dies may pose a challenge in low-batch production scenarios. The fixed costs of die maintenance, storage, and refurbishment remain constant regardless of the production volume, leading to a higher cost per part for smaller production runs. Additionally, the lead time associated with die manufacturing and setup further exacerbates the financial inefficiencies of low-batch production. The time and resources required for die design, machining, and validation may lead to prolonged production cycles and increased production costs for small quantities of parts. As a result, the cost-effectiveness of deep drawing dies diminishes for low-batch or bespoke production scenarios, where the economies of scale associated with mass production cannot be fully realised.

In recent years, the Additive Manufacturing (AM) industry has undergone significant advancement, driven by forecasts of reduced production costs and manufacturing times, as well as the proliferation of available materials for processing. This convergence of factors has opened doors for the widespread implementation of AM technology across various fields. Consequently, the exploration of integrating AM technology into sheet metal-processing industries has emerged as a novel alternative, garnering increasing attention for its potential applications.

The exploration of stamping dies fabricated by means of the AM concept has garnered attention from multiple researchers in recent times, with the preliminary findings showing promising results [8].

Attempts to manufacture a metal die made through the Selective Laser Sintering (SLS) and Sheet Lamination methods have concluded that this, although possible, is not justified by the production and post-processing costs [9,10].

Researchers in the field have turned their attention to Material Extrusion (MEX) as a manufacturing process for stamping devices due to its widespread use and accessibility. Attempts have been made to obtain embossing or bending devices using different polymers, with results that vary depending on the thickness of the processed sheet metal or the metal from which it was made.

Grigoraș et al. [11] highlighted the advantages of using AM to produce polymer dies, noting the reduced production costs and shorter manufacturing times compared to traditional metal dies. Specifically, they investigated stretch forming using dies with components 3D printed from polylactic acid (PLA). The experiments utilised six punches, varying in radius from 180 mm to 1080 mm in increments of 180 mm, to stretch aluminium 2024-T0 sheet metal strips, a material commonly used for aircraft skins. Besides evaluating the shape of the resulting parts, the study also examines other process parameters such as punch force, part radius, and deviation from circularity.

Giorleo et al. [12] explore the advantages of using AM to produce punches for the deep drawing process. The study analyses various process parameters, including the punch fillet radius, blank material, drawing ratio, and drawing depth, to determine the optimal conditions for deep drawing using MEX-produced punches. The findings indicate that the best results were achieved when drawing aluminium blanks with a drawing ratio of 1.8.

Building on this work, Alan et al. [13] investigated the use of AM technology for producing stretch forming tools used in the low-volume production of double-curvature panels, particularly in the architectural cladding and aerospace sectors. The study demonstrates the feasibility of creating a large tool by assembling smaller, printed hollow sections using FDM technology. Additionally, the research includes a comparison of the springback and surface strains between experimental trials and forming process simulations modelled in PAMSTAMP. It is particularly notable that the anticlastic tooling solution produced with 3D printing exhibited significantly lower springback, attributed to the interaction of longitudinal and transverse residual stresses.

Further studies by Madhura et al. [14] examined the viability of using AM polymer composite tooling in the automotive industry. The study employs both experimental and numerical methods to assess the performance of glass fibre-reinforced polycarbonate (GF-PC) AM polymer tooling for stamping 1.5 mm thick HSS 590 steel sheets. The experimental phase involved conducting sheet metal stamping tests with GF-PC tooling, and evaluating its performance based on tool deformation and the accuracy of the stamped parts. Complementing the experiments, finite element simulations of the stamping process were performed, accurately replicating the observed tool deformations by incorporating anisotropic material models. These simulations also proved effective in optimising the process parameters to achieve the desired final part geometry. The findings indicate that GF-PC AM polymer composite tooling is well-suited for low-volume production.

Similarly, Nakamura et al. [15] investigated the feasibility of using plastic tools, created via MEX, in sheet metal forming processes. Both aluminium and steel sheets were subjected to V-bending and deep drawing using these plastic tools. The study found that the dimensional accuracy of sheets bent with plastic tools was inferior to that achieved with steel tools, primarily due to the lower stiffness of the plastic. This accuracy further declined with an increase in the flow stress of the sheet material. To address this issue, the stiffness of the plastic tools was enhanced by reinforcing them with steel bars, resulting in the improved dimensional accuracy of the bent sheets. Additionally, modifications to the tool shapes effectively corrected for both the springback of the sheets and the elastic deformation of the plastic tools. The research also explored the deep drawability of a cylindrical cup using a plastic die, demonstrating the practical applications and potential adjustments required for plastic tooling in metal forming processes.

In a related study, Nakamura et al. [16] examined the application of plastic punches and dies, produced via the MEX process, for the V-bending of sheet metals. The research highlights the advantages of plastic tools in terms of a lower production time and cost compared to traditional steel tools, despite the significantly lower yield stress and Young’s modulus of plastic. The study examines the dimensional accuracy of bent sheets produced using these plastic tools. To enhance the accuracy, a combination of a steel punch and a plastic die was tested. The findings reveal that while the plastic tools experience substantial elastic deformation during the forming process, the springback effect is nearly identical to that observed with steel tools. This suggests that plastic tools, despite their limitations, are effective for bending sheet metals.

Additionally, Geuke et al. [17] explored the evolution from the traditional subtractive manufacturing of dies for sheet metal forming to the emerging field of AM for tooling applications. Their research evaluates the deformation and wear characteristics of an optimised AM tool during a drawing operation using a low batch size of automotive-grade sheet material. Comparative analyses are conducted between the performance of the bio-based polymer AM tool, a solid AM die approach, and a conventionally manufactured tool. This investigation underscores the potential of AM in achieving sustainable, cost-effective tooling solutions tailored for product individualisation and small batch series production, thereby contributing to advancements in manufacturing efficiency and economic viability.

Furthermore, Tondini et al. [18] investigated the viability of additively manufactured polymer tools for metal forming applications, particularly in scenarios involving small production volumes. The study begins by detailing the materials, printing strategies, and accuracy considerations for these tools. The experimental evaluations focus on the V-bending and groove pressing of 1 mm aluminium sheets using the printed tools.

In V-bending, the tools exhibit an evolving surface topography during the initial strokes, stabilising after approximately five cycles. Geometrical accuracy is assessed through parameters such as springback angle and bend radius, with the findings highlighting the influence of elastic deflection in the tools and the impact of punch nose radius variation. The study underscores the role of the printing strategy, particularly in managing the ratio between the tool radius and the thickness of the printed solid shell relative to the less dense bulk part.

Groove pressing experiments illustrate how groove heights and angular changes due to springback affect part accuracy, emphasising the importance of tool corrections to achieve closer conformity to the nominal values. Throughout the research, repeatability is a key consideration, demonstrating the potential for iterative adjustments in tool design to enhance formed part precision.

In agreement with these results, Giorleo et al. [19] showed the application of rapid tooling in forming processes, focusing on plastic punches designed for industrial-scale applications. The study addresses the challenge of adapting Additive Manufacturing to produce tools suitable for small- to medium-sized batch production. Specifically, the research investigates the performance of plastic punches in a deep drawing process using AISI 304 blanks to manufacture cups.

Both experimental and numerical analyses are employed to evaluate the quality of the cups and the behaviour of the plastic punches. The key findings indicate that larger punch dimensions correlate with the improved geometric precision of the cups, achieving high fidelity to the drawing depth (99%), cup precision on the fillet radius (98%), and a minimal roundness error (0.53%). These results highlight the potential of plastic punches manufactured through rapid tooling for enhancing the accuracy and reliability of forming operations in industrial settings.

In agreement with these results, Günther et al. [20] focused on the application of additively manufactured functional elements within deep drawing tools. Their study introduces the initial concept of these tools and details the experimental test series conducted to validate their performance. This includes an analysis of how these tools enhance the production feasibility of deep drawn parts under conditions of small batch production, aiming to bridge the gap between traditional tooling costs and the flexibility required in modern automotive manufacturing.

Although some studies have shown that, in certain circumstances, the process is not feasible, the general outcomes of processes utilising polymer-based devices and methods demonstrate the efficacy of this method in terms of both financial considerations and manufacturing duration. Despite these advancements, challenges remain in optimising the design for improving the resulting parts’ tolerances. According to Ismail et al. [21], further research is needed to address issues related to a dimensional accuracy that deteriorates significantly due to the squeezing of MEX layers after repeated stamping cycles. This gap in the literature underscores the need for continued investigation into polymer materials and their application in die manufacturing. This paper aims to demonstrate theoretically the feasibility of using polymer-based additively manufactured stamping devices instead of traditional metal ones for small batches of products and the influence of different polymers on the dimensional precision of the part products made by them. The authors are using a preliminary finite element model in order to prove such a feasibility from a theoretical point of view.

## 2. Materials and Methods

This section depicts the deep drawing process of a stamping die designed following the rules of a conventional stamping die made out of D2 tool steel. It will consequently investigate a series of comparisons with the results of the same process of several dies made out of polymeric materials. A Finite Element Analysis (FEA) study will be carried out, which will analyse the behaviour of critical die parts for each material investigated. Furthermore, the analysis processes will be divided into 4 variants, in which the thickness of the processed sheet metal will vary between 0.25 mm and 1 mm with increments of 0.25 mm.

### 2.1. Part Design

Since the design of an embossing die is closely related to the shape of the part made by it, it is essential that the part is initially designed.

**Figure 1:**
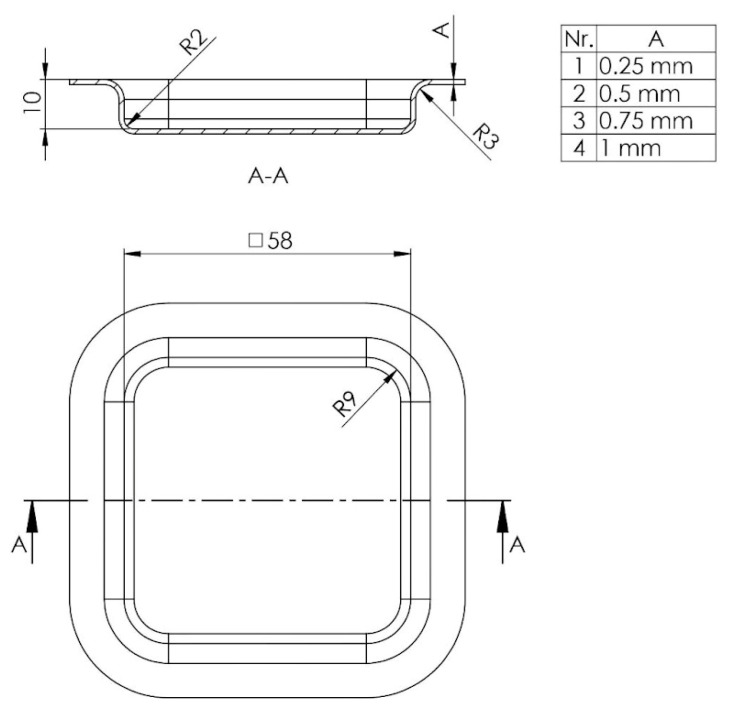
Embossing part dimensions.

Therefore, geometric characteristics like the shape and dimensions of a part subjected to the embossing process are established in this section. In order to better understand the phenomena, the chosen form is a generic one frequently encountered in industry that can have a general applicability. For the theoretical analysis of the deep drawing process, the shape of the part is a rectangular cap type with rounded corners (Figure 1).

To determine the geometric characteristics of the required tool, the minimum size of the blank stock must firstly be calculated.

Since in the process of plastic deformation the volume of the metal remains constant, the main rule for determining the dimensions of blanks for stamping processes is based on equalising the volumes of the blank and the finished part. While embossing without thinning the walls, changes in material thickness are usually neglected, and, accordingly, the determination of the dimensions of the blank is made from the condition of equality of the surface of the blank and the part, taking into account the allowance for trimming [2].

In order to determine the minimum dimensions of the semi-finished product when bending with several connection radii, the following equation is used:

(1) $$L=L_{1}+L_{2}+\cdots +L_{n}+\frac{\pi }{2}\left(r_{1}+x_{1}S\right)+\frac{\pi }{2}\left(r_{2}+x_{2}S\right)+\cdots +(r_{n-1}+x_{n-1}S)$$

where:

L—length of the flat portion;
r—inner radius of the bend;
x—coefficient with which the position of the neutral layer is determined;
S—thickness of the sheet metal.

**Figure 2:**
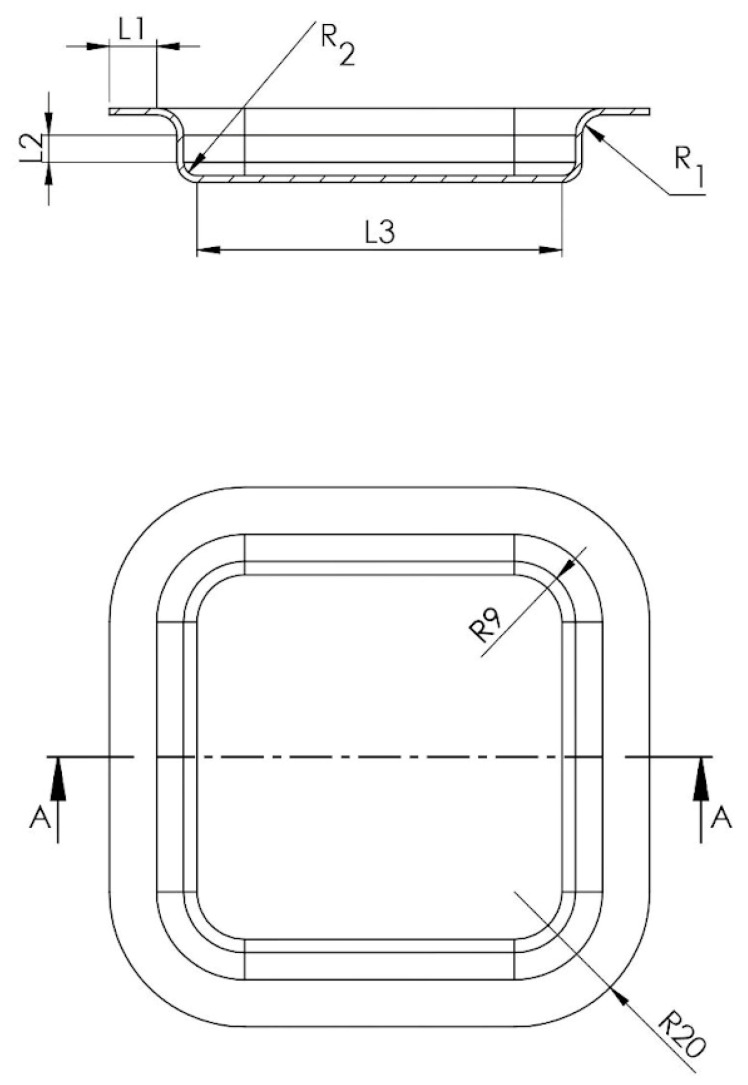
Embossing part segments.

$L_{1}$ represents the trimming edge to be cut in a process subsequent to the embossing stage, and this dimension has been conventionally assigned (Figure 2).

Table 1 presents the values of the x coefficient for the 90° bending of steel sheets [2].

Also, the length of the neutral layer in the bent portion must be determined using the equation (for φ = 90°):

(2) $$l=\frac{\pi }{2}\left(r+xS\right)$$

where:

l—length of the neutral layer in the bent position;
φ—angle of the bent portion;
r—inner bending radius;
x—coefficient with which the position of the neutral layer is determined;
S—thickness of the sheet metal.

Thus, the calculation equation for the blank side length will become:

$$\left(L_{1}\times 2\right)+\left(L_{2}\times 2\right)+L_{3}+1.57\left(R_{1}+x_{1}S\right)\times 2+1.57\left(R_{2}+x_{2}S\right)\times 2$$

where:

$L_{1}$ = 7 mm
$L_{2}$ = 4 mm
$L_{3}$ = 54 mm

thus resulting in:

$$\left(7\times 2\right)+\left(4\times 2\right)+54+\left(5.44\times 2\right)+\left(3.84\times 2\right)=94.56\;\mathrm{mm}$$

A processing allowance (20 mm/side) is inserted at this minimum length; thus, the resulting blank stock product is square in shape with a side of 120 mm.

After the dimensions of the blank have been determined, the design process of the stamping die can be started

### 2.2. Tool Design

Modelling the Computer-Aided Design (CAD) part of the device involves designing the active parts of the die such as the forming plate, blank holder, and punch; the other components that make up an embossing mould do not have a significant influence on the analysis data, and as such they will not be designated.

#### 2.2.1. Punch

**Figure 3:**
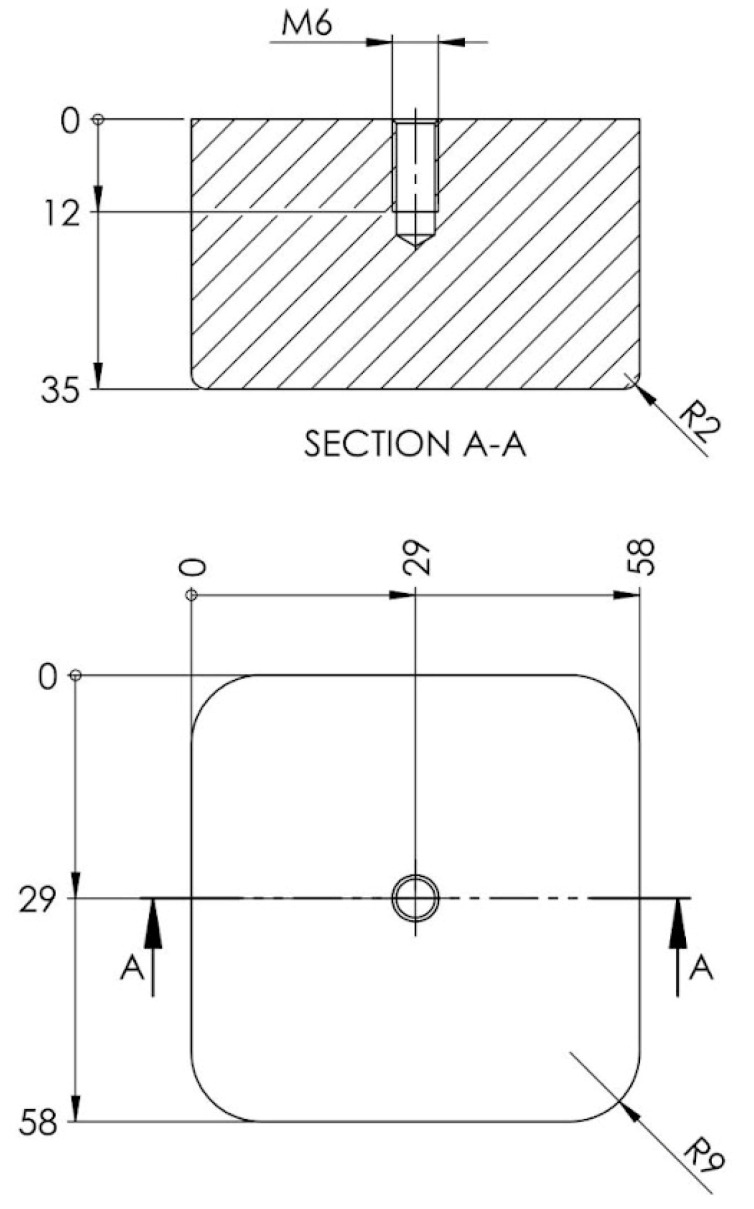
Punch dimensions.

The punch is a key component in the embossing device, exerting pressure on the blank and imprinting it with the desired shape. In the design process, the inner shape of the embossed area is used to determine the shape of the punch; thus, the punch becomes a cuboidal parallelepiped with connecting radii present on the faces that come into contact with the blank, as depicted in Figure 3. Two of its dimensions are imposed by the shape of the blank, thus becoming a square with a side of 58 mm, its height being conventionally assigned on the condition that it exceeds the height of the pressure plate when the device is operated (the die is closed). The space that is created this way between the upper plate and blank holder has the purpose of preventing damage to the blank holder by possible scratches that could appear on the embossed piece. If the pressing force is not high enough to prevent the appearance of creases on the part, it can be opted to shorten the punch at a later stage.

#### 2.2.2. Blank Holder

**Figure 4:**
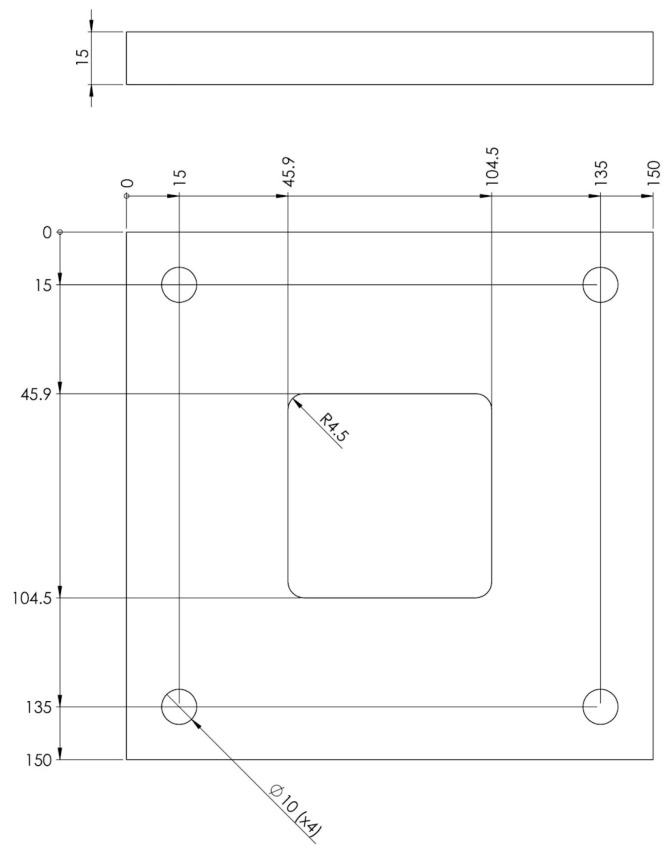
Blank holder dimensions.

The blank holder has the role of applying a uniform pressure exerted by the spring cushion on the plate during the forming process. Its absence leads to the appearance of creases and wrinkles on the blank due to the deformation forces that occur inside it during the process. Its surface is a square with a side of 150 mm to be able to completely cover the sheet metal, and its thickness has been assigned conventionally and can be modified if this is required (Figure 4). This will be provided with the guide area of the punch, where a translational clearance will be assigned to allow it to slide.

#### 2.2.3. Forming Plate

**Figure 5:**
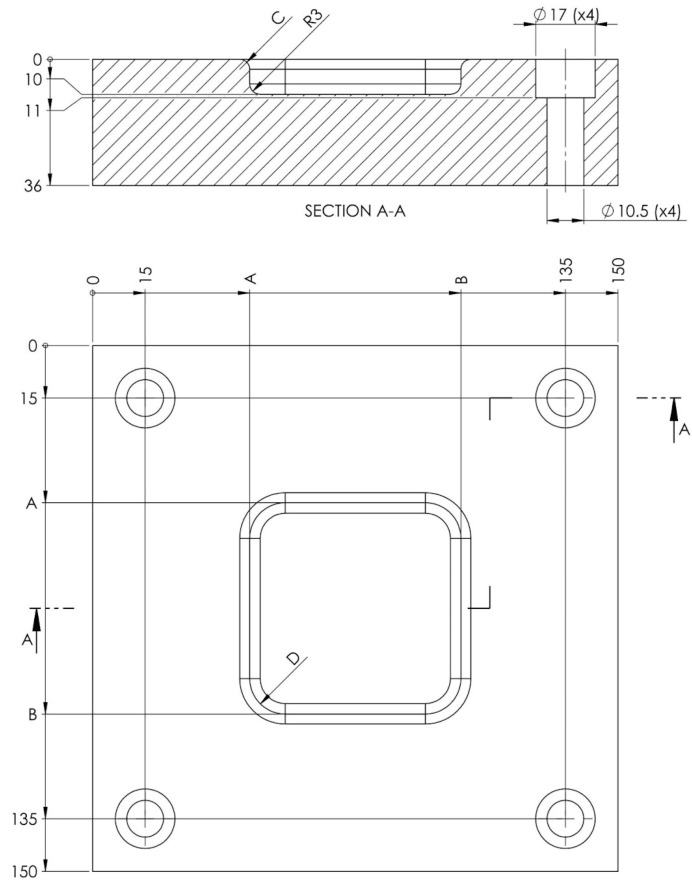
Forming plate dimensions.

The forming plate (Figure 5) has the role of providing the mechanical support for the deformation of the sheet metal piece; thus, the shape of the semi-finished product depends on its shape. The thickness of the forming plate was assigned in accordance with a conventionally made counterpart.

Given the fact that most of the embossing forces found in the device will be concentrated in the forming plate, its thickness is increased for a greater resistance to mechanical stress. The connection radii of the edges of the embossing plate have a great influence on the embossing process, such as the stresses in the embossing material and the embossing force, the value of the admissible embossing coefficient, and the formation of tears and folds. The connection radius of the embossing plate has an influence on the embossing force, so decreasing the connection radius leads to an increase in embossing force. If the radius of connection of the edges of the forming plate is increased, the embossing process is improved, because the stresses in the dangerous section are reduced, thus increasing the embossing depth possible in a single operation. Increasing the radius of the die connection, however, leads to the reduction of the surface of the part under the pressing element; thus, wrinkles and undulations can appear when the flange of the part comes out from under the retaining ring [2]. However, given the fact that the sheet metal will take the forming plate geometry, the connection radii could not be increased in order to reduce the stamping forces.

As the shape of the semi-finished product is directly correlated with the shape of this plate, a number of forming plates equal to the number of sheet thicknesses processed will be designed. The dimensions, which vary according to the thickness of the processed blank, are denoted by the letters A, B, C, and D.

Functional clearances are provided between the punch and the forming plate to reduce the friction between the die and the material (Table 2). When determining the size of the clearance, the increase in the thickness of the blank at the edges during embossing and the non-uniformity of the material thickness must be taken into account. It should be emphasised that a single clearance value cannot be established, considering the fact that the parts to be stamped differ greatly in terms of dimensions, precision, technical conditions, and the stamping process used (without holding pressure, with pressing or turning the edges). A small value of the clearance causes an increase in the resistance to embossing, which leads to an increase in tensile stresses in the dangerous section and a decrease in the degree of deformation.

Thus, for each thickness of the blank, a forming plate will be designed to which a specific addition is assigned to the processed material, the punch and the pressing plate remaining common (Table 3).

The model designed this way presents the necessary characteristics that will allow it to be FEA-studied and manufactured later by means of MEX technology, and to be assembled, together with the other components that will define the whole assembly of the stamping device.

### 2.3. FEA

Ansys is a suite of engineering simulation software developed by Ansys Inc. It uses numerical methods such as FEA to solve complex engineering problems and provide insights into product performance, reliability, and safety [22].

Thus, Ansys Workbench 2022 R1 was used to complete this stage, where a simulation of the behaviour of a conventionally made steel device was carried out, and the results were compared against a series of such devices made of polymers.

The first step involved the model simplification, where redundant features (such as bolt holes and connecting radii) were removed, which could have prevented a fast calculation.

The rest of the process consists of assigning the simulation parameters, such as the material properties of the device and the blank, establishing the connections between parts, generating the mesh of the model, and then defining the joint movements, to simulate the kinematics of the stamping die during the deep drawing process.

#### 2.3.1. Model Simplification

SpaceClaim 19.0, a solid modelling CAD software integrated in Ansys as a built-in 3D modeller, was used for the model simplification. It uses a direct modelling approach based on solid modelling, where design features are created by pulling, moving, filling, combining, and reusing 3D shapes [23]. The fill function was employed to fill the gaps in selected regions or to delete certain elements, and the parameters for the analysis were configured for the thus-simplified model.

#### 2.3.2. Materials

In order to simulate a conventionally manufactured device, the active elements of the die were assigned as D2 steel material, frequently used in industry due to properties that give it a very high resistance to wear [3], and the sheet metal chosen was DIN 1.1121 steel, due to mechanical properties that recommend it for the process.

Some of the most common materials available to the MEX process (ABS, PLA, PET, and Nylon) were used to simulate the devices made via the AM method. The material model employed in the simulation was the elastic–plastic model, with bilinear isotropic hardening to simulate the die and anisotropic plasticity models for the sheet metal, which has directional properties from rolling.

#### 2.3.3. Connections

For the part contacts, the frictional type was selected, whose coefficient of friction was 0.03, in order to simulate a process in which lubrication is considered at the contact between the active parts made of steel and the sheet metal [24]. For the polymer devices where lubrication is not needed [15,20], a coefficient of 0.04 was assigned [24]. To simulate the stamping process, the bending plate was fixed at the bottom (similar to the clamping on a base plate), and similarly, the pressure plate was fixed at the top to simulate the constant application of the pressing force via the spring cushion.

#### 2.3.4. Mesh Generation

Given the general parallelepipedal shape of the parts, mesh elements with a cuboid shape can be assigned in order to obtain a regular and uniform distribution of elements. Thus, the results can be obtained in a much shorter period of time with a smaller probability of errors incurred during the solving time.

After comparing the results of several iterations in which the element was assigned different dimensions, it was concluded that the size of 2 mm offered the optimal balance between the precision of the results and the computation time. So, for the parts meshing process, the Hex-dominant method was chosen with an element size of 2 mm, to obtain the most uniform distribution of the elements with a parallelepipedal shape. In regions with complex geometry or high stress gradients (connection radii of the forming plate), additional techniques such as local mesh generation strategies and the use of mesh control tools were employed for creating a uniform and good-quality mesh in critical areas, ensuring that the mesh accurately captures both the stress gradients and geometric details.

#### 2.3.5. Displacement

Since the kinematics of the die is completely vertical, the movement of the punch can be considered a translational movement that lacks any rotational joints.

As such, the punch displacement was implemented with Joint—Displacement, assigning a translation over a distance of 10 mm (corresponding with the final part height) and returning it to the starting position in 4 steps, where each individual step takes 1 s. Thus, a stroke of the punch is achieved with a constant speed of 5 mm/s.

## 3. Result

Several simulations were carried out, where five devices made of different materials were tested, performing a deep drawing process on four metal sheets of different thicknesses. In total, 20 simulations were completed, and their results were centralised and then compared.

Using the results provided by total deformation, the resulting part dimension can be established.

**Figure 6:**
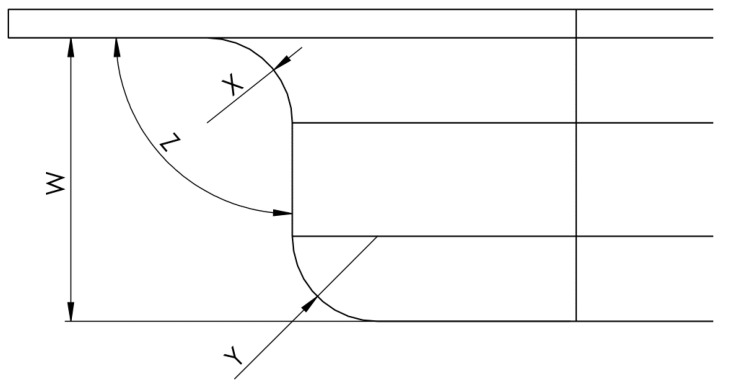
Samples area of probe.

The determination of the critical dimensions of the parts was carried out on the models in the STL format of the resulting part. Thus, the connection radii were measured, along with the angle of incidence of the part wall (see Figure 6). The dimensions thus measured were compared with the table General Tolerances to DIN ISO 2768-mk [25] to determine the influence of the material of the device on the tolerances of the parts.

According to ISO 2768-mk [25], each dimension without tolerance values is assigned a set of general tolerances depending on the desired precision. Thus, the general tolerances for linear and angular dimensions that are classified in four accuracy categories—f (fine), m (medium), c (coarse), and v (very coarse)—were collected for the dimensions of interest (Table 4, Table 5 and Table 6). This was done to have a reference regarding the tolerances offered by the dies made of different polymers.

The desired length of the W dimension is 10 mm, and this represents the height of the part. A negative deviation from this translates into the insufficient compression resistance of the stamping die.

The desired size of the X dimension is 3 mm and that of the Y dimension is 2 mm + the sheet thickness. These are the connecting radii of the part, and a positive deviation from this translates into a high elasticity coefficient of the material of the stamping die.

The Z dimension should be as close as possible to the value of a right angle (90°), and its increase represents the weak resistance of the device to the compression forces involved in the processing of the material.

Coarse deviations can be noticed in the case of parts resulting from devices made of polymers by comparison with those made from classic devices (Table 7). However, these linear deviations are small and can be corrected during the design process of the device. Angular deviations are more significant, PLA and PET being the materials that most satisfy these conditions.

As in the case of the 0.25 mm plate processing devices, coarse deviations for linear dimensions can also be observed here (Table 8). Also, for angular dimensions, the devices made of PLA and PET showed the best behaviour.

A thickness of 0.75 mm for the processed sheet raises problems for the devices made of ABS and Nylon (Table 9), because the tolerances offered are no longer found in the ISO standard, and the resulting parts may be classified as non-compliant. In the case of devices made of PLA and PET, the tolerances offered tend to become very coarse.

**Figure 7:**
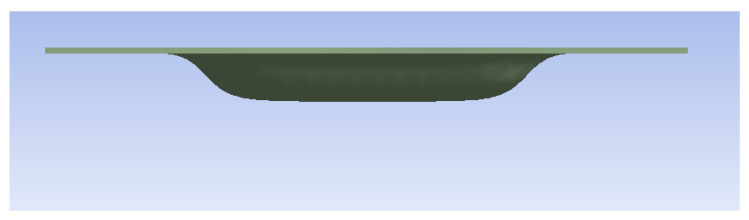
Example of rejected part (ABS for 1 mm sheet metal).

**Figure 8:**
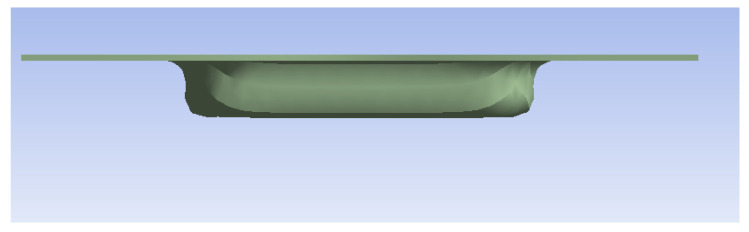
Example of part deemed as fit (D2 steel for 1 mm sheet metal).

A thickness of 1 mm pushes the resistance of devices made of ABS and Nylon to their limit (Table 10), the resulting parts being categorically non-compliant with the proposed requirements (Figure 7). Also, devices made of PLA and PET make parts deemed as inappropriate; the only device capable of making compliant parts is the one made of steel (Figure 8).

Differences can be observed between the parts obtained on conventionally made devices and the parts obtained on devices made of polymers.

Thus, an initial comparison is made (Table 11), where the biggest differences are highlighted between the results obtained by means of polymer devices compared to those made conventionally.

Using the results provided by total deformation, the impact of the displacements on different parts of the die during and after the deep drawing process can be determined.

The remaining deformations in the forming plate will be translated as deviations from the desired shape of the next processed part—implicitly, the capacity of reusing the device a second time.

The results provided by equivalent stress underline the capacity of the devices to withstand the process, depending on the material from which they are manufactured and the loads to which they are subjected. In FEA, equivalent stress, often referred to as von Mises stress, is a scalar value used to predict the yielding of materials under complex loading conditions. It is derived from the stress state at a point and provides a single value that reflects the combined effect of all the individual stress components (normal and shear stresses) acting on the material. This measure is crucial in assessing whether the parts manufactured of different materials will yield or fail when subjected to the applied loads. If the equivalent stress at any point in the part exceeds the material’s yield strength, yielding is expected to occur at that point. This indicates potential failure or permanent deformation.

**Figure 9:**
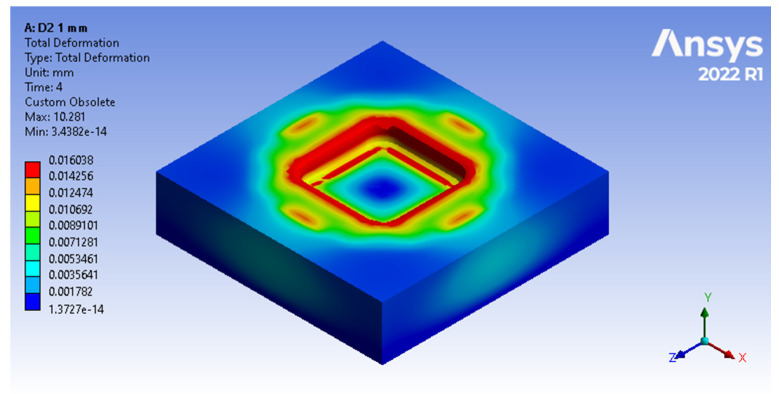
Total deformations of the forming plate incurred during the embossing process, as viewed through Ansys Workbench window.

**Figure 10:**
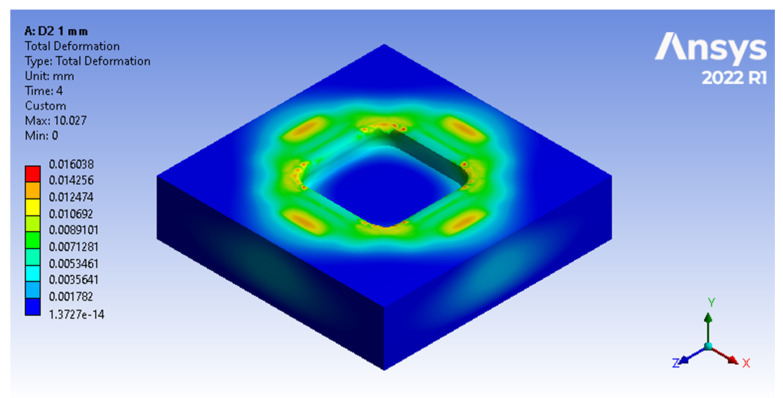
Total deformations of the forming plate that persist after the embossing process, as viewed through Ansys Workbench window.

For the results captured through total deformations (Figure 9 and Figure 10), the material displacements in the device are showcased by a colour gradient, where the largest material displacements are indicated with a red colour and the unaffected areas are indicated in blue.

**Figure 11:**
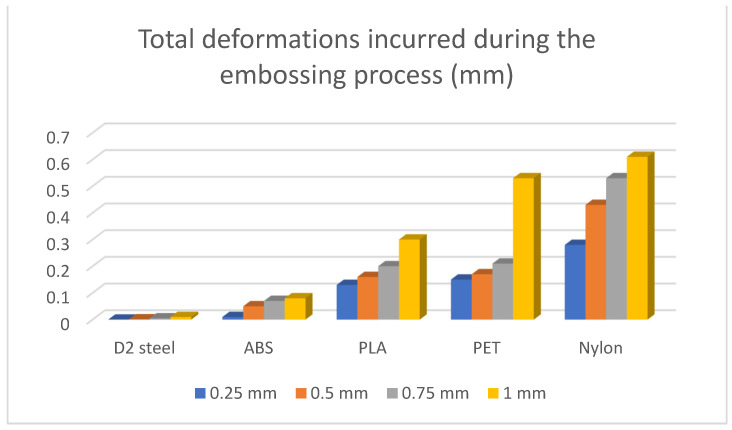
Total deformations of the forming plate incurred during the embossing process (mm).

**Figure 12:**
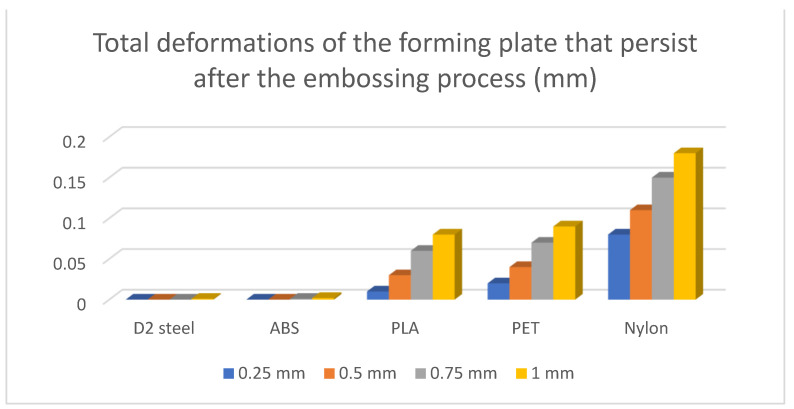
Total deformations of the forming plate that persist after the embossing process (mm).

The results of the simulations are summarised (Table 12 and Table 13, Figure 11 and Figure 12) in the form of the highest indicated value based on the type of material and the thickness of the processed sheet.

In Figure 9, it can be observed how the tension concentrates at the radii of the forming plate, and how this is the most affected area. For a similar analysis between devices made of different materials, the measuring area is chosen as the straight area between them, as that determines the gauge dimensions of the part. It is also worth mentioning that the results highlight the propagation of stresses along and in the depth of the plate.

A series of events can be observed such as that the devices made of steel show an insignificant deformability in the process of embossing metal sheets. It is also possible to observe the good behaviour of the devices made by MEX, results that allow one to use such devices in the embossing process.

These deformations are imprinted on the part, and they influence deviations from its imposed external dimension.

The result of the analysis shows which are the most affected areas of the forming plate (Figure 10) by compression following the embossing process.

The return to the initial shape of devices made of ABS presents a similar characteristic to that of devices made conventionally. A similarity of the behaviour is also observed between the devices made of PLA and PET, while the devices made of Nylon show the weakest characteristic of returning to the initial shape.

These deformations are imprinted to the next part that will be processed on it. With these results, we can determine the mechanical characteristics of the devices, such as their ability to process a larger number of parts or their life cycle.

**Figure 13:**
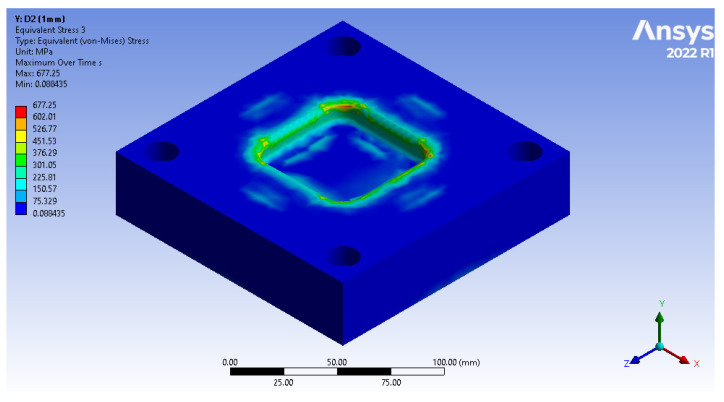
Maximum equivalent stress incurred in forming plate, as viewed through Ansys Workbench window.

For the results captured through equivalent stress (Figure 13), the highest stress of one of the principal directions for each element is illustrated through a chromatic spectrum, where blue represents the areas that store the lowest stress. The stress increases progressively, altering the colour gradually, depending on its intensity. The red colour marks the areas where the greatest concentration of forces is found, as well as the areas most susceptible to failure due to mechanical forces.

The details regarding the mechanical properties of the materials (Table 14) were taken from the Ansys Workbench database, and they will be compared with the nominal values obtained during the analysis.

Following the preliminary results (Table 15), the devices made of PLA, PET, and Nylon present an equivalent stress that exceeds the tensile yield stress; however, those areas are found in a narrow area of the device. This can translate into irreparable deformations of certain areas at the corners of the device. This phenomenon is predictable and occurs at the first embossing of the device, the following cycles not affecting the condition of the die in a significant way [15,20].

**Figure 14:**
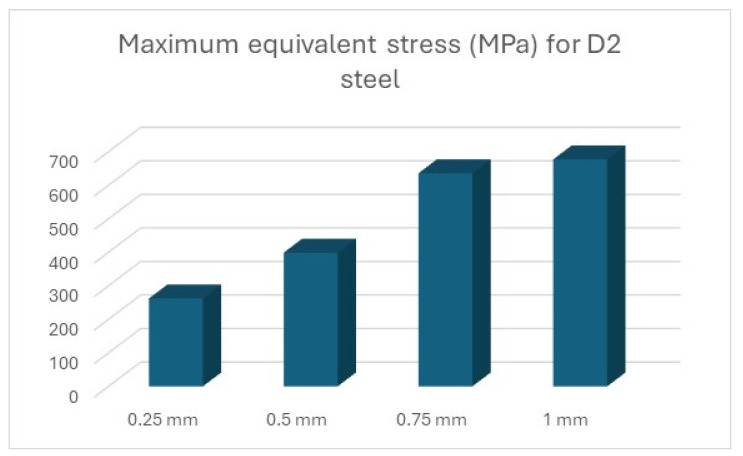
Maximum equivalent stress for D2 steel.

Thus, the graphic representation of the maximum equivalent stress for the devices was completed, where the equivalent stress that appears in the forming plate during the processing of sheets of different thicknesses can be observed and compared to the stress that can be seen in the forming plate made of steel (Figure 14).

**Figure 15:**
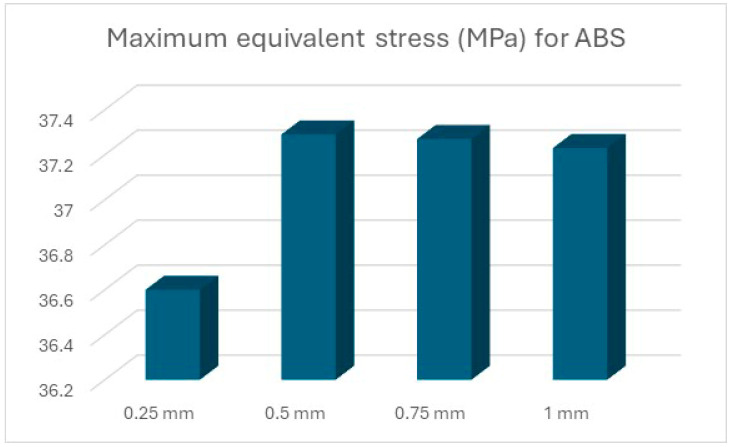
Maximum equivalent stress for ABS.

In the case of the devices made through the AM process, the lowest stress area can be found in the parts made of ABS (Figure 15). This material showed a limit behaviour even for thin sheet metalprocessing applications, with a stress value close to the stress limit. The stress varies slowly in respect to the shell thickness. For this material, a bilinear hardening model was employed. Bilinear isotropic hardening is a material model which allows the progressive expansion of the elastic limit. The yield strength increases as plastic deformations occur [26]. Another problem with ABS is that the material is time-dependent, both in elasticity and plasticity [27]. Although ABS proved to have the best behaviour regarding the handling of equivalent stress, further investigations are required for the acceptance of this material as punch or die parts.

**Figure 16:**
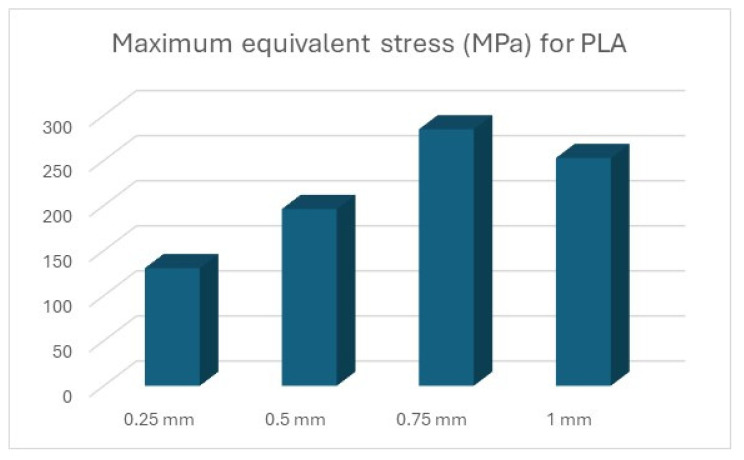
Maximum equivalent stress for PLA.

**Figure 17:**
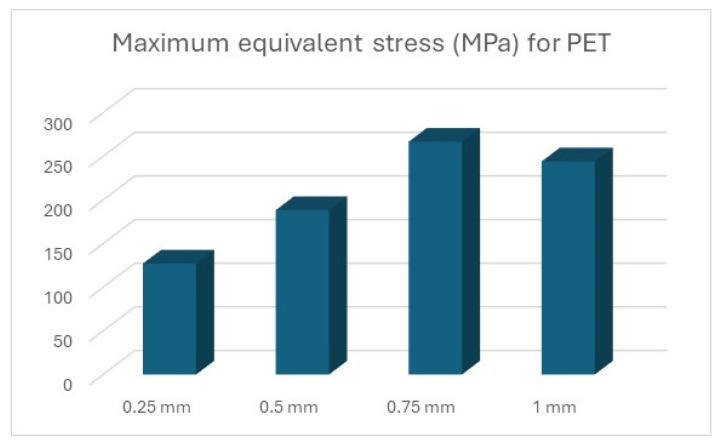
Maximum equivalent stress for PET.

**Figure 18:**
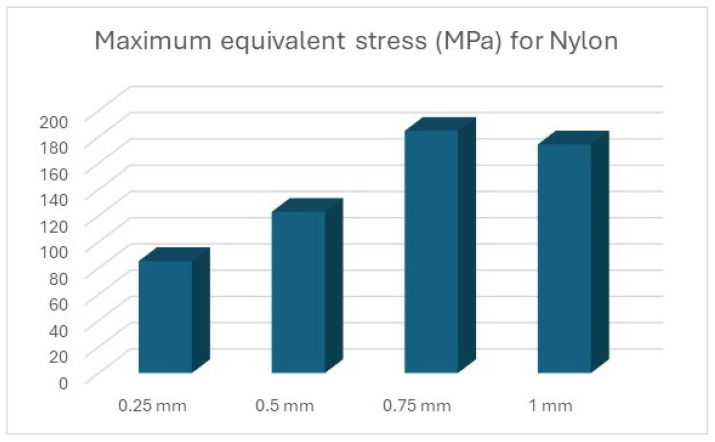
Maximum equivalent stress for Nylon.

In the case of PLA (Figure 16), PET (Figure 17), and Nylon (Figure 18), exceeding the values for tensile ultimate strength can be observed, meaning permanent changes in the shape of the device. However, these areas are limited to the radius on which the metal sheet is drawn, not being present in its entire structure.

The results provided by the total deformations analysis are accompanied by the equivalent stress analysis, providing a complete picture of the behaviour of polymers in the embossing process of metal sheets.

## 4. Discussion

These results highlight the effectiveness and potential of polymeric die sets in deep drawing applications, specifically focusing on materials like PLA and PET. The results indicate that these materials demonstrate superior wear resistance and minimal deformation, which is attributed to their higher tensile strength and impact resistance compared to Nylon and ABS. Notably, the study found that PLA and PET had acceptable differences in part production when compared to conventional dies, especially with thin sheets. For instance, the deviations observed were 0.3 mm for 0.25 mm sheets and 0.15 mm for 0.5 mm sheets.

Further analysis showed that devices made from PLA and PET exhibited areas exceeding maximum equivalent stress values, particularly in the radii regions during the embossing process. This aligns with previous findings by Nakamura et al. [15] and Günther et al. [20], suggesting that subsequent embossing processes would result in minimal deformation, indicating the potential for iterative tool design improvements. On the other hand, Nylon devices performed adequately for thinner sheets (0.25 mm)—not as effectively as PLA and PET, yet better than ABS.

ABS, however, demonstrated significant limitations due to its flexural strength properties, resulting in poor shape definition on metal sheets and substantial deviations in rays, ranging from 4.22 degrees for 0.25 mm thick sheets to 45 degrees for 1 mm sheets. This suggests ABS’s limited applicability for precise deep drawing operations but highlights the potential for its use in less demanding applications or where flexibility and recovery are critical.

In summary, while polymeric materials like PLA and PET show promise for deep drawing applications, offering a balance of strength, wear resistance, and manufacturability, further research and optimisation are required to enhance their dimensional accuracy and application scope. This study underscores the necessity for continued investigation into the materials’ properties and their practical implications in die manufacturing to fully leverage their potential in industrial settings. This study can be continued by streamlining the process, using a range of special materials, or improving mechanical characteristics by inserting standardised objects (screws, metal bars) or non-standardised ones (plates made of other materials, or elements specially designed for their respective case) into the forming plates in order to minimise the deformations occurring within the process, to enhance the precision of parts and to increase the lifetime of devices.

## 5. Conclusions

This study investigated the use of polymeric materials, specifically PLA, PET, Nylon, and ABS, for manufacturing die sets in deep drawing processes. The results demonstrate the potential of PLA and PET as viable alternatives to conventional steel dies, particularly for applications involving thin metal sheets.

The key findings include the following:

- PLA and PET exhibited superior wear resistance and minimal deformation compared to Nylon and ABS. These materials maintained acceptable deviations in part production, with measurements of 0.3 mm for 0.25 mm thick sheets and 0.15 mm for 0.5 mm thick sheets.
- Areas of maximum equivalent stress were identified in the radii regions during the embossing process, with PLA and PET showing minimal deformation in subsequent processes, corroborating the findings of Nakamura et al. and Günther et al.
- Nylon performed adequately for 0.25 mm thick sheets but was less effective for thicker sheets, while ABS showed significant limitations due to its flexural strength properties. ABS resulted in poor shape definition and substantial deviations in rays, indicating its limited applicability for precision deep drawing operations.

The study concludes that PLA and PET offer promising characteristics for use in deep drawing applications, providing a balance of strength, wear resistance, and manufacturability. However, further research is needed to enhance their dimensional accuracy and broaden the application scope of these materials. Continued investigation into their material properties and practical implications in die manufacturing is essential to fully exploit their potential in industrial settings.

## Figures and Tables

**Table 1:** The values of the x coefficient.

$\frac{r}{S}$ Ratio	x Coefficient
0.1	0.3
0.2	0.33
0.25	0.35
0.3	0.36
0.4	0.37
0.5	0.38
0.6	0.385
0.8	0.405
1	0.42

**Table 2:** Clearance value related to metal sheet thickness [2].

Sheet Metal Thickness (mm)	Clearance (mm)
0.2	0.05
0.5	0.1
0.8	0.12
1	0.15
1.2	0.17
1.5	0.19
1.8	0.21
2	0.22

**Table 3:** Resulting dimensional values variation of forming plate.

Value (mm)	0.25 mm	0.5 mm	0.75 mm	1 mm
A	45.7	45.4	45.13	44.85
B	104.3	104.6	104.87	105.15
C	2.95	2.9	2.88	2.85
D	9.3	9.6	9.87	10.15

**Table 4:** Permissible deviations in mm for ranges in nominal lengths for linear dimensions.

Linear Dimensions (mm)	f (Fine)	m (Medium)	c (Coarse)	v (Very Coarse)
Over 6 up to 30	±0.1	±0.2	±0.5	±1.0

**Table 5:** Permissible deviations in mm for ranges in nominal lengths for external radius and chamfer heights.

External Radius (mm)	f (Fine)	m (Medium)	c (Coarse)	v (Very Coarse)
0.5 up to 3	±0.2	±0.2	±0.4	±0.4

**Table 6:** Permissible deviations in degrees and minutes for ranges in nominal lengths for angular dimensions.

Angular Dimensions (°)	f (Fine)	m (Medium)	c (Coarse)	v (Very Coarse)
Over 50 up to 120	±0°20′	±0°20′	±0°30′	±1°

**Table 7:** Resulted dimensions for 0.25 mm.

Material	W (mm)	X (mm)	Y (mm)	Z (deg)
D2 steel	9.89	3.6	2.5	90
ABS	9.6	3.72	3.25	93.6
PLA	9.63	3.61	3.2	91.51
PET	9.59	3.62	3.2	91.52
Nylon	9.19	3.69	3.51	94.22

**Table 8:** Resulted dimensions for 0.5 mm.

Material	W (mm)	X (mm)	Y (mm)	Z (deg)
D2 steel	9.73	3.3	3.08	90
ABS	9.64	5.07	5.05	108.86
PLA	9.69	3.46	3.94	93.85
PET	9.58	3.85	4.32	94.05
Nylon	9.28	3.76	4.37	101.31

**Table 9:** Resulting dimensions for 0.75 mm.

Material	W (mm)	X (mm)	Y (mm)	Z (deg)
D2 steel	9.73	3.5	3.3	90
ABS	9.17	8.14	7.14	127.17
PLA	9.58	3.7	3.9	96.3l
PET	9.5	4.04	4.33	97.47
Nylon	9.17	4.42	4.32	103.19

**Table 10:** Resulted dimensions for 1 mm.

Material	W (mm)	X (mm)	Y (mm)	Z (deg)
D2 steel	9.72	3.59	3.7	90
ABS	9	10.09	10.95	135
PLA	9.68	3.94	4.03	98.75
PET	9.53	4.15	4.69	100.02
Nylon	8.97	4.93	4.8	110.86

**Table 11:** Greatest dimension differences between parts made through conventional and polymer devices.

Sheet Thickness	W (mm)	X (mm)	Y (mm)	Z (deg)
0.25 mm	0.7	0.12	1.01	4.22
0.5 mm	0.45	1.77	1.97	18.86
0.75 mm	0.56	4.64	3.84	27.17
1 mm	0.75	6.5	7.25	45

**Table 12:** Horizontal deformations of the forming plate incurred in the embossing process (mm).

Material	0.25 mm	0.5 mm	0.75 mm	1 mm
D2 steel	0	0.001	0.005	0.01
ABS	0.01	0.05	0.07	0.08
PLA	0.13	0.16	0.2	0.3
PET	0.15	0.17	0.21	0.32
Nylon	0.28	0.43	0.53	0.61

**Table 13:** Total deformations of the forming plate that persist after the embossing process (mm).

Material	0.25 mm	0.5 mm	0.75 mm	1 mm
D2 steel	0	0	0	0.001
ABS	0	0	0.001	0.002
PLA	0.01	0.03	0.06	0.08
PET	0.02	0.04	0.07	0.09
Nylon	0.08	0.11	0.15	0.18

**Table 14:** Tensile yield strength and tensile ultimate strength for studied materials.

Material	Tensile Yield Strength (MPa)	Tensile Ultimate Strength (MPa)
D2 steel	2066	2292
ABS	36.13	38.73
PLA	52.44	62.93
PET	52.44	57.45
Nylon	38.86	49.75

**Table 15:** Maximum equivalent (von Mises) stress achieved during embossing process (MPa).

Material	0.25 mm	0.5 mm	0.75 mm	1 mm
D2 steel	262.32	399	635.8	677.25
ABS	36.6	37.29	37.27	37.23
PLA	130.61	196.22	284.58	252.75
PET	127.2	188.47	266.75	244.08
Nylon	85.32	122.83	184.55	174.22

## Data Availability

The data that support the findings of this study are available from the corresponding author upon reasonable request. The data are not publicly available due to privacy.
